# HIV-1 Productively Infects and Integrates in Bronchial Epithelial Cells

**DOI:** 10.3389/fcimb.2020.612360

**Published:** 2021-02-04

**Authors:** Dinesh Devadoss, Shashi P. Singh, Arpan Acharya, Kieu Chinh Do, Palsamy Periyasamy, Marko Manevski, Neerad Mishra, Carmen S. Tellez, Sundaram Ramakrishnan, Steven A. Belinsky, Siddappa N. Byrareddy, Shilpa Buch, Hitendra S. Chand, Mohan Sopori

**Affiliations:** ^1^ Department of Immunology and Nano-Medicine, Herbert Wertheim College of Medicine, Florida International University, Miami, FL, United States; ^2^ Respiratory Immunology Division, Lovelace Respiratory Research Institute, Albuquerque, NM, United States; ^3^ Department of Pharmacology & Experimental Neuroscience, University of Nebraska Medical Center, Omaha, NE, United States; ^4^ Lung Cancer Program, Lovelace Respiratory Research Institute, Albuquerque, NM, United States; ^5^ Department of Surgery, University of Miami Miller School of Medicine, Miami, FL, United States

**Keywords:** lung epithelial cells, HIV receptors, genomic integration, HIV *gag-pol*, Nested-PCR, Fluorescent *in-situ* hybridization

## Abstract

**Background:**

The role of lung epithelial cells in HIV-1-related lung comorbidities remains unclear, and the major hurdle in curing HIV is the persistence of latent HIV reservoirs in people living with HIV (PLWH). The advent of combined antiretroviral therapy has considerably increased the life span; however, the incidence of chronic lung diseases is significantly higher among PLWH. Lung epithelial cells orchestrate the respiratory immune responses and whether these cells are productively infected by HIV-1 is debatable.

**Methods:**

Normal human bronchial epithelial cells (NHBEs) grown on air–liquid interface were infected with X4-tropic HIV-1_LAV_ and examined for latency using latency-reversing agents (LRAs). The role of CD4 and CXCR4 HIV coreceptors in NHBEs were tested, and DNA sequencing analysis was used to analyze the genomic integration of HIV proviral genes, Alu-HIVgag-pol, HIV-nef, and HIV-LTR. Lung epithelial sections from HIV-infected humans and SHIV-infected macaques were analyzed by FISH for HIV-gag-pol RNA and epithelial cell-specific immunostaining.

**Results and Discussion:**

NHBEs express CD4 and CXCR4 at higher levels than A549 cells. NHBEs are infected with HIV-1 basolaterally, but not apically, by X4-tropic HIV-1_LAV_ in a CXCR4/CD4-dependent manner leading to HIV-p24 antigen production; however, NHBEs are induced to express CCR5 by IL-13 treatment. In the presence of cART, HIV-1 induces latency and integration of HIV provirus in the cellular DNA, which is rescued by the LRAs (endotoxin/vorinostat). Furthermore, lung epithelial cells from HIV-infected humans and SHIV-infected macaques contain HIV-specific RNA transcripts. Thus, lung epithelial cells are targeted by HIV-1 and could serve as potential HIV reservoirs that may contribute to the respiratory comorbidities in PLWH.

## Introduction

In the era of combined antiretroviral therapy (cART), the life span of PLWH has increased considerably; however, the incidence of obstructive lung diseases (OLDs) is significantly higher in PLWH (Maitre et al., 2018; Singhvi et al., 2019). A major hurdle in curing HIV is the persistence of latent HIV reservoirs in PLWH (Richman et al., 2009; Vanhamel et al., 2019). While CD4+ memory T cells are the recognized HIV reservoirs (Chun et al., 1997), recent evidence has demonstrated the presence of a minor macrophage subpopulation, which harbors HIV-1 in cART-treated HIV-infected subjects (Andrade et al., 2020). Lungs are established reservoirs of HIV-1 (Almodovar, 2014); however they may also be one of the preferred HIV sanctuaries in cART-treated subjects. Thus, bronchoalveolar lavage (BAL) is enriched in memory T cells and BAL cells contain 13-fold higher HIV-DNA than peripheral blood mononuclear cells (Costiniuk et al., 2018). The role of epithelial cells as HIV-1 targets is ambiguous. For example, the foreskin epithelial cells are known to express HIV coreceptors (Liu et al., 2014), and upon coculturing, transfers the HIV to lymphocytes (Yasen et al., 2017). NHBEs express CXCR4 and respond to X4-tropic gp120 treatment (Gundavarapu et al., 2013), and herein, we show that they also express CCR5 after IL-13 treatment; however, whether these cells are productively infected by HIV-1 remains debatable (Brune et al., 2016; Chinnapaiyan et al., 2017). Previously, we reported that lung tissues of cynomolgus macaques (CMs) infected with simian-adapted HIV (SHIV) had significantly higher HIV-gp120-positive epithelial cells (Chand et al., 2018); however, the source of gp120 was not established. In this study, we demonstrate that HIV-infected NHBEs, grown on the air–liquid interphase (ALI) produce HIV-p24, and the surviving HIV-1-infected cells harbor the HIV-provirus integrated within the host cell genome, which is activated by latency-reversing agents (LRAs). Moreover, *ex-vivo* analysis of lung epithelial cells from HIV-infected human subjects and SHIV-infected macaque lungs harbor HIV-specific RNA, suggesting a productive infection of lung epithelial cells by HIV-1 in both *in-vitro* and *in-vivo*.

## Materials and Methods

### Normal Human Bronchial Epithelial Cells and ALI culture

Normal Human Bronchial Epithelial cells (NHBEs) were obtained from MatTek Incorp (EpiAirway™, Ashland, MA) and cultured as described previously (Hussain et al., 2018). The apical surface of the cells was exposed to air, and the cells were incubated in the media (provided by MatTek) for 48 h at 37°C before infection with HIV-1.

### HIV-Infected Lung Tissue Sections

The archived lung tissue sections were kindly provided by the National NeuroAIDS Tissue Consortium (NNTC) and Lung Tissue Research Consortium (LTRC) of NIH. The formalin-fixed and paraffin-embedded human patient samples were from HIV-1-negative (HIV−) donors and HIV-1-positive (HIV+) subjects, and from HIV+ subjects on HAART (HIV+HAART). The lung tissue sections from SHIV-infected (SHIV+) and cigarette smoke (CS)-exposed SHIV+ macaques (CS+SHIV) were obtained from the study reported previously (Chand et al., 2018).

### HIV Infection and p24 Analysis

The X4-tropic viral strain HIV-1_LAV_ was employed in these studies. NHBEs grown in air–liquid-interface (ALI) in transwells were infected either basolaterally (BL) or apically (AP) with X4-tropic HIV-1_LAV_ (5 ng/ml p24 equivalent). Control cultures received only the medium. Control and infected transwells were incubated for 2 h at 37°C in 5% CO_2_ atmosphere. At 2 h post-infection, BL-infected cells were washed 4× with 2 ml PBS and the apically AP-infected cells with 0.5 ml PBS. The fourth wash from all cultures was analyzed for HIV-1-p24 level to ensure removal of the residual virus; this time point was designated as 0-time. Fresh medium was added to the cultures, and the plates were incubated at 37°C in 5% CO_2_; the cultures were harvested at the indicated times.

The p24 concentration in the transwell culture media was measured using a p24 capture ELISA kit as per manufacturer’s instructions (ZeptoMetrix Corp. Cat # 0801200 or Advance Bioscience Laboratories, Rockville, MD, USA). To promote latently in HIV-infected AECs, following infection of NHBEs with HIV-1, where indicated, the cells were transferred to the medium containing cART (a cocktail of ritonavir, tenofovir, and emtricitabine) for 6 days. The cells were washed, transferred to the culture medium without cART and, where indicated, treated with the latency-reversing agents (LRAs) vorinostat and LPS, as described before (Archin et al., 2012; Alvarez-Carbonell et al., 2017). At 24 h after the transfer, culture media were harvested and assayed for p24 levels.

### Immunostaining and Fluorescent Imaging Analysis

For immunohistochemical staining, deparaffinized and hydrated lung tissue sections were washed with PBS containing 0.05% Brij-35 (pH 7.4) and immunostained for antigen expression as described previously (Chand et al., 2018). Briefly, the antigens were unmasked by steaming the sections in 10 mM citrate buffer (pH 6.0) followed by incubation in a blocking solution containing 3% BSA, 1% Gelatin, and 1% normal donkey serum with 0.1% Triton X-100 and 0.1% Saponin; the sections were stained with antibodies to CD4 (Abcam, #ab133616), CXCR4 (Abcam, #ab181020), HIV-Tat (Abcam, #ab63957) and pan-CK (#4545, Cell Signaling Technologies, Danvers, MA). Similarly, to stain NHBEs, the cells were grown on coated coverslips or the coated Labtek-II slides (Thermo Fisher Inc.) and fixed in 4% paraformaldehyde. The slides were washed with PBS containing 0.05% Brij-35 (pH 7.4) and immunostained as described previously (Chand et al., 2017). Briefly, non-specific binding sites on the cells were blocked by a solution containing 3% BSA, 1% Gelatin, and 1% normal donkey serum with 0.1% Triton X-100 and 0.1% Saponin. The cells were then treated with antibodies to CD4, CXCR4, and pan-CK, as described above. The labeled cells/tissue sections were stained with appropriate second antibodies conjugated with fluorescent dyes (Jackson ImmunoResearch Lab Inc., West Grove, PA). The slides were treated with 4′,6-diamidino-2-phenylindole (DAPI) containing Fluormount-GTM (SouthernBiotech, Birmingham, AL) to visualize cell nuclei. Immunofluorescent images were captured using BZX700 Microscopy system (Keyence Corp., Japan) and analyzed using NIH Image J software.

### DNA Isolation

To isolate DNA from the transwell-grown NHBEs, cells were dislodged from the filters and digested in 2.0 ml Eppendorf tubes containing 1.5 ml of digestion buffer (pH 8.0) comprising SDS, EDTA, and proteinase K as per manufacturer’s instructions (MatTek Corp, MA, USA). Total DNA from the control and HIV-1 treated NHBEs was isolated using Qiagen DNA extraction kit (Germantown, MD, USA) and quantified using Nanodrop (Applied Biosystems, ThermoFisher).

### HIV-1 Gag DNA Quantification

The HIV-1 infected NHBEs were collected from the transwells at different time points (0, 2, 4, 24, 48, and 96 h) after infection. DNA was isolated from the cells using Qiagen DNA extraction kit per manufacturer’s instructions (QIAGEN Germantown, MD, USA), and cell associated HIV-1 gag DNA was quantified as described by Pasternak et al. (2008). using Applied Biosystems QuantStudio 3 Real-Time PCR System (Applied Biosystems, Waltham, MA, USA), and the data was used to calculate HIV-1 gag copies/ng of DNA.

### Alu-Gag PCR and Nucleotide Sequencing

We utilized the methods as described previously (Liszewski et al., 2009) in which a two-step Alu-gag PCR assay was used to determine the presence of integrated HIV-1 proviral DNA. This method utilizes a nested PCR approach. Briefly, during the 1^st^ round PCR, the region between HIV-1 gag gene and nearest Alu repeat element of host genome was amplified. The primer sequences used in the 1^st^ step PCR were: Alu Forward: 5′-GCCTCCCAAAGTGCTGGGATTACAG-3′ and HIV gag reverse: 5′-GTTCCTGCTATGTCACTTCC-3′ which corresponds to nucleotide (nt) 1,505–1,486 of HIV-1 HXB2 genome. The reactions were carried out in 50 µl and contained 1.5 mM MgCl_2_, 0.2 mM dNTPs mix, 100 nM Alu Forward primer, 600 nM HIV gag reverse primer and 5 U of Platinum Taq DNA polymerase (Life Technologies; USA). In the 2^nd^ round PCR, the 5′ LTR region of HIV-1 was amplified using 1 µl of the 1^st^ round PCR amplicon as the template. The primers for the second round were: 5′ LTR Forward: 5′-TTAAGCCTCAATAAAGCTTGCC-3′ and 5′ LTR Reverse: 5′-GTTCCTGCTATGTCACTTCC-3′. The PCR amplification was carried out in Applied Biosystem 9700 thermal cycler under the following conditions, 1^st^ round of PCR: 95°C for 2 m, followed by 40 cycles at 95°C for 15 s, 50°C (T_m_) for 15 s, and 72°C for 3.5 min; 2^nd^ round of PCR: 95°C for 2 m, followed by 40 cycles at 95°C for 15 s, 60°C (T_m_) for 15 s and 72°C for 30 s. PCR amplicons were resolved on a 2% agarose gel (Promega Corporation, Madison, USA) pre-stained with Ethidium bromide (0.5 μg/ml). The gel images were documented using a Bio Rad gel documentation system (BIORAD, USA).

The PCR amplicons were purified using QIAGEN PCR purification kit (QIAGEN, Germany) as per the manufacturer’s instructions and subjected to Sanger sequencing using BigDye Terminator v3.1 Cycle Sequencing Kit (Applied Biosystems, California; USA). Automated capillary electrophoresis was performed on an ABI PRISM^®^ 3500 Genetic Analyzer (Applied Biosystems, California; USA) using data collection software v.3.1 at the University of Nebraska Medical Center DNA Sequencing Core facility. The raw sequence data were manually edited, spliced, and assembled by Sequencher v4.9 to generate the final contig. Multiple sequence alignment of edited sequence was performed with HIV-1_LAV_ reference sequence of HIV-1 using Clustal W (Aiyar, 2000).

### HIV-1 Gag DNA Amplification and Sequencing

HIV-1 gag DNA from infected NHBEs was amplified essentially using the primers, probes, and PCR protocol described previously (Khoja et al., 2008). Briefly, primers used in first round of PCR on cellular DNA were forward primer (5′-CTCTCGACGCAGGACTCGGCTTGC-3′, nt 683–706, HXB2) and reverse primer (5′-CCAATTCCCCCTATCATTTTTGG-3′, nt 2,382–2,404) with a T_m_ of 58°C. For the second round of amplification, forward primer (5′-GAGGCTAGAAGGAGAGAGATGGG-3′, nt 772–794, HXB2) and GIPR (5′-TTATTGTGACGAGGGGTCGTTGCC-3′, nt 2,269–2,292) with a T_m_ of 60°C were used. The amplicon (1,520 bp) was analyzed on 1.2% agarose gels following ethidium bromide staining. For sequencing, the amplified DNA product was extracted from the agarose gel, and purified, using QIAquick Gel Extraction kit (Cat#28704, Qiagen Inc.) based on the manufacturer’s instructions. The product was sent for sequencing to Sequetech Corp (Mountain View, CA).

### HIV-1 Nef DNA Amplification and Sequencing

Nef DNA was also amplified by a two-step nested PCR strategy as described previously (Shugars et al., 1993). Briefly, the two sets of amplification primers were designed to anneal to highly conserved segments flanking the HIV-1 nef gene. The primers used for the first round of amplification were, forward primer (5′-AATAGAGTTAGGCAGGGATA-3′, nt 8,338-8,358, HXB2) and reverse primer (5′-CTGGTCTAACCAGAGAGACCCAGTAC-3′, nt 9,533-9,558) with a T_m_ of 55°C. For the second round of amplification, forward primer (5′-CTCGCAGTCTAGAAGAATAAGACAGGGCTTGGAAAGG-3′, nt 8,754-8,782, HXB2) and reverse primer (5′-CGTCCAGAATTCGGAAAGTCCCCAGCGGAAAGTC-3′, nt 9,436–9,457) were used that included the restriction sites for XbaI and EcoR1, respectively and amplified at the T_m_ of 55°C. The PCR product from the first round of amplification (1,200 bp) was diluted 1:100 for the second round of amplification using 30 PCR cycles. The PCR product (703 bp) was visualized by ethidium bromide staining and agarose gel electrophoresis. The amplified Nef DNA was extracted from the agarose gel, purified using QIAquick Gel Extraction Kit, and sent for sequencing to Sequetech Corp (Mountain View, CA).

### Quantitative Real-Time RT-PCR

Total RNA was isolated from the experimental cells using RNAeasy kit (Qiagen, Germantown, MD) as per manufacturer’s instruction. RNA concentration was determined using the Synergy HTX Multi-Mode reader (BioTek, Winooski, VT), and cDNA was synthesized using iScript advanced cDNA kit (BioRad, Hercules, CA). The primer/probe sets for CD4, HIV-LTR RNA, and MUC5AC were obtained from Applied Biosystems (Thermo Fisher Inc.). The amplified cDNA was quantified by qPCR using the TaqMan Gene expression kit (Thermo Fisher Inc.) in the Stratagene Mx3000P Real-Time PCR System (Agilent, Santa Clara, CA). Relative quantities were calculated by normalizing the averaged C_T_ values to CDKN1B or GAPDH to obtain ΔC_T_, and the fold-change (ΔΔC_T_) over the controls was determined as described previously (Chand et al., 2017).

### Lactate Dehydrogenase Release Assay

The loss of plasma membrane integrity is considered as a maker of cell death and is measured as the release of cytosolic LDH in culture media after the rupture of plasma membrane. The concentration of LDH in the culture supernatants collected from HIV-1 infected NHBE transwells was measured by using CytoTox96 assay (Promega, Madison, WI) as per manufacturer’s instructions.

### RNA Fluorescent *In-Situ* Hybridization

RNA FISH was essentially performed using the RNAscope^®^ Fluorescent Multiplexed reagent kit (Advanced Cell Diagnostics, Newark, CA) as per the manufacturer’s protocol and as reported recently (Devadoss et al., 2020). Briefly, the probe set for HIVgag-pol consisted of 20 dual probes targeting different segments within the whole transcript (Advanced Cell Diagnostics). Deparaffinized and permeabilized lung sections were hybridized with probes for 2 h at 40°C using a HyBEZ^®^ oven (Advanced Cell Diagnostics), and the signal was amplified by serial incubation in amplification buffers and HRP-tagged probe (Thermo Fisher Inc), at 40°C using a HyBEZ^®^ oven. Probes were detected using Tyramide signal amplification (TSA) reaction using an Alexa-flour-labeled TSA kit (PerkinElmer Bioscience) according to the manufacturer’s instructions. The sections were processed for immunostaining of panCK as described above, followed by mounting with 4′,6-diamidino-2-phenylindole (DAPI) containing Fluormount-G (SouthernBiotech, Birmingham, AL) to visualize the nuclei. Immunofluorescence images were captured with BZX700 Microscopy system (Keyence Corp, Japan) and analyzed by NIH ImageJ software. RNA FISH expression was quantified by the analysis and as reported recently (Devadoss et al., 2020). Briefly, signals (dots/cell) for each transcript probe were counted and allocated to separate bins with Bin 0 (0 Dots/Cell); Bin 1 (1–3 Dots/Cell); Bin 2 (4–9 Dots/Cell); Bin 3 (10–15 Dots/Cell); Bin 4 (>15 Dots/Cell). The histology score (H-Score) was calculated as Sum of each (bin number × percentage of cells per bin) that ranged from 0 to 400 based on the transcript’s expression.

### Western Blot Analysis

Cell extracts were prepared using RIPA buffer (20 mM Tris, pH 7.4, 137 mM NaCl, 1% NP-40, 0.25% Deoxycholate, 0.1% SDS, 1 mM EDTA and 1% protease inhibitor). Protein concentration was determined by BCA kit (Pierce; Rockford, IL), and 50 µg of protein was analyzed by western blotting as described previously (Hussain et al., 2018). Proteins were detected by ECL and visualized by chemiluminescence (Perkin Elmer, Waltham, MA) using the BioRad Chemidoc Imaging system (Hercules, CA).

### Statistical Analysis

Grouped results were expressed as means ± SEM. Data were analyzed using GraphPad Prism Software (GraphPad Software, Inc., San Diego, CA). Grouped results were analyzed using two-way analysis of variance. When the effects were significant (p ≤ 0.05), the Fisher’s least significant difference test was used to determine differences between groups.

## Results and Discussion

### Human Bronchial Epithelial Cells Intrinsically Express CD4 and CXCR4, and Express CCR5 Upon IL-13 Treatment

**Figure 1:**
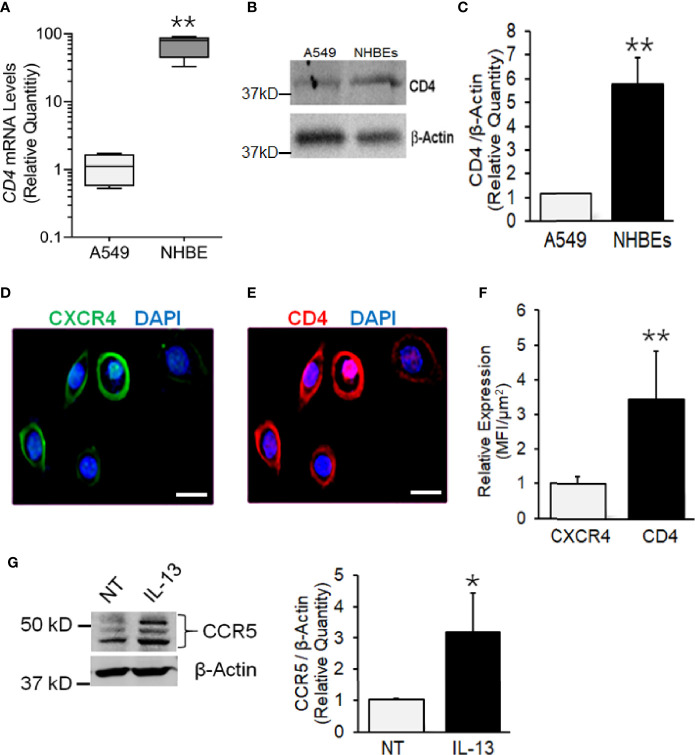
Human airway epithelial cells express HIV receptor and co-receptors. **(A)** qPCR analysis of CD4 mRNA levels in NHBEs compared to A549 cells. **(B)** Western blot analysis of CD4 protein levels in NHBE and A549 cells with β-actin as the loading control. **(C)** Densitometric analysis of CD4 protein levels compared to A549 cells. Representative micrographs of NHBE cells showing **(D)** CXCR4 (green) and **(E)** CD4 (red) immunopositivity along with DAPI-stained nuclei (blue); scale—5µ. **(F)** Relative expression of CXCR4 and CD4 in NHBEs as assessed by measuring the Mean Fluorescence Intensity (MFI) per unit area. **(G)** Western blot analysis of CCR5 protein levels in NHBE cells treated with IL-13 (1 ng/ml) for 48 h compared to non-treated (NT) control cells and densitometric analysis of CCR5 levels normalized to *β*-actin levels compared to controls. Data shown as mean ± SEM; n = 3/group and are representative of three different experiments; *p < 0.05; **p < 0.01.

CD4, CXCR4, and CCR5 are HIV-1 receptors/coreceptors (Brelot and Chakrabarti, 2018). NHBEs express CXCR4 (Eddleston et al., 2002; Gundavarapu et al., 2013), and it is X4- but not the R5-tropic HIV-gp120 that induces airway inflammatory mucus in these cells (Gundavarapu et al., 2013). Based on the qPCR and immunoblot analysis, compared to A549 (a human alveolar epithelial cell line), NHBEs showed >50-fold higher expression of *CD4* mRNA (**Figure 1A**) and >5-fold higher CD4 protein levels (**Figures 1B, C** **)**. Immunocytometry staining indicated that, in addition to CXCR4 (**Figure 1D**), NHBEs exhibited high levels of CD4 expression (**Figure 1E**), and the CD4 levels were approximately 3.5-fold higher than CXCR4 (**Figure 1F**). Thus, NHBEs express HIV-1 receptor CD4 and coreceptor CXCR4, rendering them as potential HIV-1 targets.

The expression CCR5 on NHBEs is not unequivocal (Brune et al., 2016; Chinnapaiyan et al., 2017). During carcinogenesis, epithelial cells have been shown to express CCR5 that enhances their resistance to cytotoxicity (Jiao et al., 2019). Th2 responses in the lung are common and are induced by infections and exposure to allergens (Hansbro et al., 2014). IL-13 is a key Th2 cytokine, which is strongly upregulated in the lungs of SHIV-infected macaques (Chand et al., 2018). Moreover, CCR5 plays a critical role in the IL-13-induced lung pathogenesis (Ma et al., 2006). Therefore, we determined whether IL-13 affects the expression of CCR5 on NHBE cells. As shown in **Figure 1G**, IL-13 (1 ng/ml) treatment induced the significant levels of CCR5 in NHBEs. Therefore, it is likely that *in-vivo* lung epithelial cells are primed to express CCR5.

### X4-Tropic HIV-1_LAV_ Infects NHBEs

**Figure 2:**
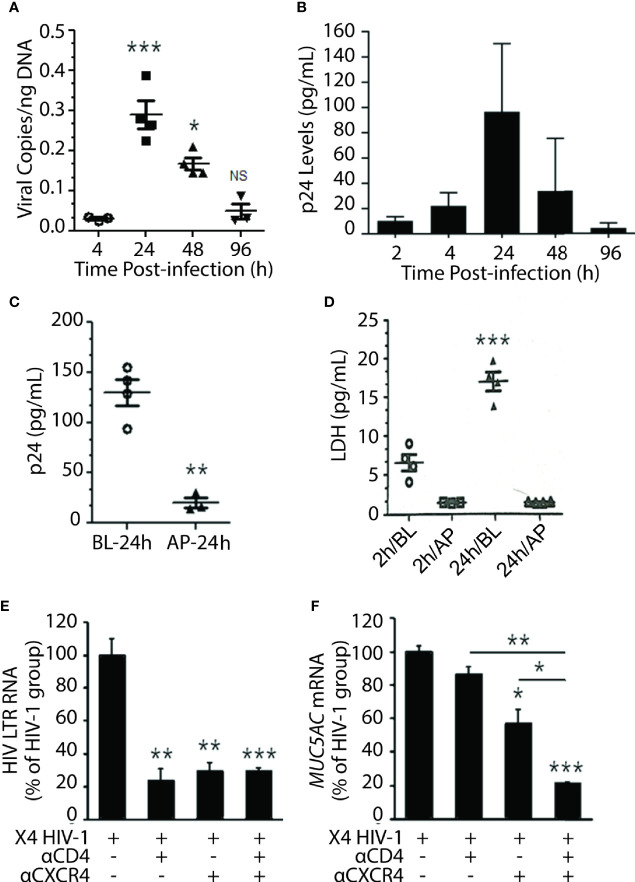
Differentiated NHBE cells were infected by X4-tropic HIV-1 and blocking HIV-coreceptors inhibits HIV infectivity. **(A)** Expression kinetics of HIV-1 gag DNA levels (viral copies per ng of cellular DNA) in NHBE cell lysates analyzed at 2, 4, 24, 48, and 96 h post X4-tropic HIV-1_LAV_ infection. **(B)** Expression kinetics of p24 (pg/ml) measured in cell culture media collected from the bottom of transwells at the denoted time post infection. **(C)** HIV-1 p24 levels at 24 h post-infection in NHBEs that were infected *via* basolateral (BL) or apical (AP) surfaces; and **(D)** LDH release quantification at 2 and 24 h post-infection in BL- or AP-infected cells. Relative quantities of **(E)** HIV-1 LTR RNA levels and **(F)** *MUC5AC* mRNA levels in NHBE cells pre-treated with either anti-CD4 or anti-CXCR4 antibodies followed by infection with X4-tropic HIV-1_LAV_ and 48 h post- infection cells were harvested and analyzed. Data shown as mean ± SEM; n = 3/group; *p < 0.05; **p < 0.01; ***p < 0.001.

Among the HIV-1 patients, the R5 (CCR5-tropic) HIV-1 variants predominate over the course of infection, and the X4 (CXCR4-tropic) variants arise later (Poon et al., 2012); however, nearly 22% of patients on cART principally harbor the X4 variants (Ferrer et al., 2014). Moreover, a recent study indicated that X4 variants are mainly associated with pulmonary arterial hypertension—a lung condition quite prevalent among PLWH (Almodovar et al., 2020). Most importantly, NHBEs explicitly express CXCR4 (Eddleston et al., 2002; Gundavarapu et al., 2013), and the X4- but not the R5-tropic HIV-gp120 induces inflammatory mucus response in NHBEs (Gundavarapu et al., 2013). Therefore, to ascertain whether NHBEs are productively infected by HIV-1, ALI-differentiated NHBEs were infected with X4-tropic HIV-1_LAV_. Cellular DNA was isolated and assayed for HIV-gag by qPCR at denoted time points (**Figure 2A**), and the culture supernatants were analyzed for HIV-p24 levels (**Figure 2B**). Both HIV-1 titer (HIV-1 cell-associated DNA) and p24 levels reached maximum at 24 h and declined thereafter to very low levels at 96 h post-infection. HIV-1 infection induces cell death (Cooper et al., 2013), and HIV-1 infection of NHBEs showed increased levels of the cell-death marker lactate dehydrogenase (LDH) (**Figure S1**).

Transmission of HIV-1 *via* oral or oropharyngeal route is highly unlikely (CDC Report August 6, 2019: www.cdc.gov/hiv/basics), and the saliva may inactivate HIV-1 (Baron et al., 1999). We analyzed the susceptibility of NHBEs to HIV-1 infection *via* basolateral (BL) *versus* AP apical (AP) side. NHBEs grown on ALI were infected with HIV-1 from either AP or BL surfaces, and p24 levels were measured at 24 h. LDH levels assessed at 2 and 24 h post-infection indicated that NHBEs are mainly sensitive to HIV-1 from BL side and relatively resistant to HIV infection from AP side (**Figure 2C**); and BL infection with HIV-1 showed increased LDH release (**Figure 2D**). Thus, the apical side of differentiated lung epithelial cells is essentially refractory to HIV-1 infection.

Next, to verify that basolateral HIV-1 infection is mediated *via* HIV receptors/coreceptors, NHBEs were pre-incubated with anti-CXCR4 and/or anti-CD4 antibodies. The HIV-LTR RNA levels were potently inhibited in cells pre-incubated with antibodies to CD4 and/or CXCR4 compared to control HIV-1 infected cells (**Figure 2E**). Similarly, HIV-1-induced MUC5AC mucin expression was significantly reduced in cells pretreated with CD4- and/or CXCR4-antibodies (**Figure 2F**). These data suggest that HIV-1 infection of bronchial epithelial cells is dependent on classical HIV-1 receptors/coreceptors.

### HIV-1 Establishes Latency With Proviral DNA Integration Into NHBE Genome

Although HIV-1-induced cell death is associated with genomic integration of HIV-1 DNA (Cooper et al., 2013); the proviral integration in BECs remains uncertain. Treatment with antiretroviral drugs suppresses HIV-1 below detection limits but also promotes infected-cell survival and establishes HIV-1 latency (Dahabieh et al., 2015). The latent cells harbor replication-competent integrated HIV-1 provirus; however, the cells remain transcriptionally silent establishing them as viral reservoirs (Margolis and Archin, 2017; Sengupta and Siliciano, 2018). LRAs activate latent HIV-1 proviruses, leading to the production of the virus (Takahama and Yamamoto, 2020). We observed that the treatment of NHBEs with cART (a cocktail of ritonavir, tenofovir, and emtricitabine) at 0-time (*i.e.*, 2h after HIV-1 infection), inhibited but failed to eliminate p24 expression at 8 h post-infection (**Figure S1B**).

**Figure 3:**
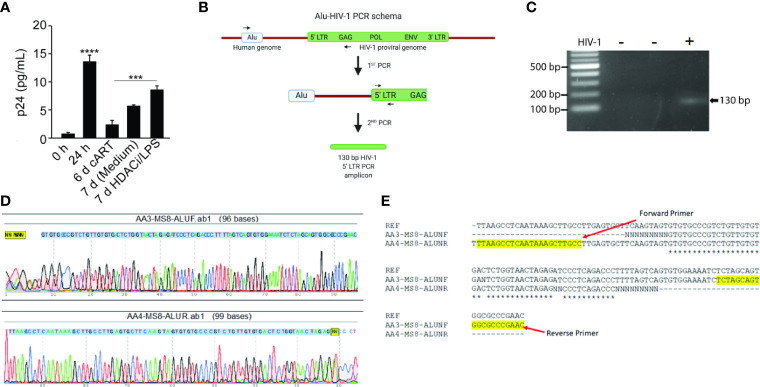
Differentiated NHBE cells act as HIV-1 reservoir and Alu-PCR analysis confirming the integration of HIV-1 proviral genome in NHBEs. **(A)** HIV-1 p24 levels after latency-reversing agents, vorinostat and LPS, treatment of HIV-1 infected cells. **(B)** Schematics of the nested-PCR approach used to confirm the HIV DNA integration in infected NHBEs. **(C)** Agarose gel analysis of the nested PCR amplicon showing amplification of 130 bp band from 5′ LTR region of HIV-1. **(D)** Electropherogram of the 130 bp PCR amplicon generated using Forward and Reverse primers confirms the presence of the HIV proviral genome (The 5′-LTR sequence of HIV is indicated with blue shade). **(E)** Multiple sequence alignment of edited HIV-1 5′-LTR sequence obtained from the sequencing of the 130 bp PCR amplicon along with the sequence of HIV-1_LAV_ used to infect the NHBEs. The primer sequences used are highlighted in yellow. Data shown as mean ± SEM; n = 3/group; ***p < 0.001; ****p < 0.0001.

To demonstrate that HIV-1 infects and establishes latency, NHBEs were infected with HIV-1_LAV_ at 24 h after HIV-1 infection and the culture media were replaced with cART-containing media. Infected NHBEs were culture d for 6 more days, washed, and replenished with the culture media without cART and treated with the LRAs comprised of LPS, a gram-negative bacterial wall component and Vorinostat, a histone deacetylase inhibitor (HDACi) (Archin et al., 2012), After 24 h, the culture supernatants were harvested and assayed for p24 levels. As shown in **Figure 3A**, before the removal of cART, cells had very low levels of p24 (day 6 with cART); however, at 24 h after cART removal, there is a small but significant increase in p24 levels (7 day media control). Media harvested from cells treated with LRAs (7 day-HDACi/LPS) had significantly higher p24 levels suggesting that, in the presence of cART, NHBEs are latently infected by HIV-1, and the latency is reversed by LPS and vorinostat. Together, these results suggest that in the presence of cART, HIV-1 infection of NHBEs yields latently infected cells and the latent HIV-1 provirus is activated by LRAs, leading to the reemergence of proviral factors.

For productive infection and proviral latency, HIV-1 DNA needs to be integrated into the host genome (Craigie and Bushman, 2012). To demonstrate that HIV-1 DNA is integrated into NHBEs, we analyzed genes in 5′- and 3′-LTR of HIV (*i.e.* gag and Nef genes, respectively). DNA isolated from control and HIV-infected NHBEs was used to amplify HIV-gag in a nested PCR, and there was a single 1.52 kb amplicon present in the infected cells (**Figure S2A**). The amplicon was isolated and sequenced (**Figure S2B**); the sequence alignment confirmed >99% identity to the HIV-1 gag sequence. Similarly, Nef gene was amplified using the nested-PCR approach that yielded a single amplicon of 703 bp (**Figure S3A**). The sequencing analysis confirmed >99% identity to HIV-1 Nef sequence (**Figure S3B**). In the second approach, a two-step Alu-gag PCR assay was employed (**Figure 3B**). The two rounds of PCRs resulted in a 130 bp amplicon of 5′-LTR of HIV-1 genome (**Figure 3C**), and the amplicon was sequenced (**Figure 3D**). The sequence alignment of the raw sequencing data exactly matched with the reference sequence of LTR-gag region of HIV-1 strain used for the infection (**Figure 3E**), confirming the chromosomal integration of HIV provirus in NHBEs. Taken together, these data support the presence of several HIV-1 genes in the cellular DNA from HIV-infected NHBE cells and the association of Alu sequences with the LTR DNA. Alu sequences are present only in primate DNA (Quentin and Fichant, 1994) and represent at least 20% of human genome (Schmid, 1996). Thus, the association of Alu sequences with HIV-specific LTR DNA clearly indicates that HIV is integrated within the NHBE cellular DNA.

### HIV RNA Present in Lung Epithelial Cells From SHIV-Infected Macaques and HIV-Infected Human Subjects

**Figure 4:**
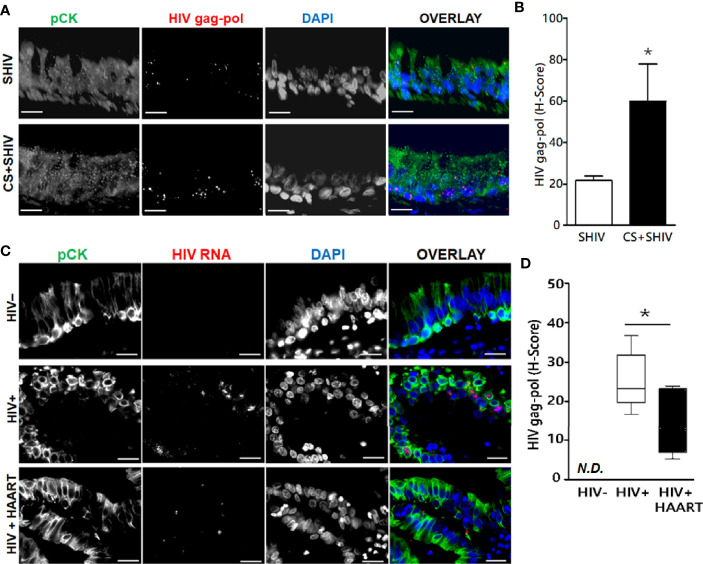
Expression of HIV-gag-pol RNAs in the lung epithelial cells of SHIV-infected macaques and HIV-infected human subjects treated with antiretrovirals. Archived 5 μm FFPE lung tissue sections were used for HIV-1-gag-pol RNA detection using RNAscope^®^ and immunostained for the epithelial cell marker, pan-cytokeratin (pCK). **(A)** Representative micrographs of bronchial epithelial cells showing pan-cytokeratin, pCK (green), and HIV-gag-pol (red) colocalization along with the DAPI-stained nuclei (blue) from SHIV- and CS+SHIV-infected groups; CS, cigarette smoke, Scale—5µ. **(B)** Quantification of epithelial HIV-gag-pol expression denoted as H-score, see *Materials and Methods* for details; *p < 0.05. **(C)** Representative micrographs of bronchial epithelial cells showing pCK (green) and HIV-gag-pol (red) colocalization along with the DAPI-stained nuclei (blue) in archived lung tissues from HIV-negative controls (HIV−), and from HIV-infected (HIV+) subjects and HIV+ subjects on HAART treatment (HIV+HAART). Scale—5µ. **(D)** Quantification of HIV-gag-pol expression in bronchial epithelial cells (denoted as H-score). N.D., Not detected. Data are shown as mean ± SEM; n = 4/group; *p < 0.05.

To ascertain whether HIV replicates *in-vivo* in airway epithelial cells, we evaluated lung sections for HIV-specific RNA by FISH using RNAScope^®^ and HIV-gag-pol probes (ACD Biotechne Inc.). HIV-specific gag-pol RNA showed 20-fold higher expression in SHIV-infected pan-cytokeratin (pCK)-positive BECs than uninfected control lungs (**Figures 4A, B** **)**. Moreover, SHIV infection of cigarette smoke (CS)-exposed macaques (CS+SHIV) showed about three-fold higher levels of HIV-specific RNA than HIV-infected controls, thus, supporting the evidence that combined exposure of CMs to CS and SHIV is more injurious to the lung than independent exposures to either SHIV or CS (Chand et al., 2018). Similarly, the lung sections from HIV-infected subjects showed >25-fold higher levels of HIV-specific RNA in pCK-positive epithelial cells, and the high levels of the RNAs persisted (14-fold higher than controls) in HIV subjects undergoing HAART (HIV+HAART) compared to uninfected human airway control sections (**Figures 4C, D** **)**. These results underscore the inference that both in humans and in macaques, the lung epithelial cells are targets of HIV-1 infection and might be responsible for the higher respiratory comorbidities among PLWH.

Lung is an important reservoir for HIV-1 (Almodovar, 2014; Costiniuk et al., 2018), and herein, we demonstrate that the airway epithelial cells are targets of HIV-1 infection in both *in-vitro* and *in-vivo* settings and that in the presence of cART the cells harbor latent HIV-1 provirus. Lung bronchial epithelial cells are crucial innate immune cells and the first line of defense against airborne pathogens (Wu et al., 2011). In the respiratory tract, airway epithelial cells are constantly exposed to airborne pathogens, including LPS-containing gram-negative bacteria (Hayes et al., 2013), and we have shown that LPS, a signature molecule in gram-negative bacteria, in conjunction with Vorinostat, can activate the latent HIV-provirus in HIV-infected airway epithelial cells. Given that airway epithelial cells are very long-lived (Rawlins and Hogan, 2008), in the presence of cART, these cells are likely to make good targets for latent HIV infections and may significantly contribute to the HIV-1-reservoir function of the lung.

## Data Availability Statement

The original contributions presented in the study are included in the article/**Supplementary Material**; further inquiries can be directed to the corresponding author.

## Ethics Statement

All experimental protocols carried out on cynomologus macaques were approved by the Institutional Animal Care and Use Committee of Lovelace Respiratory Research Institute, Albuquerque, NM in accordance with the Guidelines from the Association for the Assessment and Accreditation for Laboratory Animal Care International as reported recently (Chand et al., 2018).

## Author Contributions

DD and SS performed the sample analysis, analyzed the data, and wrote the manuscript. AA, KD, PP, MM, NM, and CT performed the sample analysis and analyzed the data. SR, SAB, SNB, and SB analyzed the data. HSC and MS designed the studies, analyzed the data, and wrote the manuscript. All authors reviewed the manuscript. All authors contributed to the article and approved the submitted version.

## Funding

The authors acknowledge the funding support by NIH R01HL125000 (to MS), R01DA047089 (to SR), and R21AI144374, R21AI152937, R21AI117560, R01HL147715, and the FIU Start-Up Funds (to HSC). S.A.B. is supported in part by NIH P30CA11800 grant to C. Willman.

## Conflict of Interest

The authors declare that the research was conducted in the absence of any commercial or financial relationships that could be construed as a potential conflict of interest.

## Abbreviations

ALI, Air-liquid interface; AP, Apical side; BECs, Bronchial epithelial cells; BL, Basolateral side; cART, Combined antiretroviral therapy; CD4, Cluster of differentiation 4; CMs, Cynomolgus macaques; COPD, Chronic obstructive pulmonary disease; CS, Cigarette smoke; CXCR4, C-X-C chemokine receptor type 4; FISH, Fluorescent *In-Situ* Hybridization; Gag, Group specific antigen; HAART, Highly active antiretroviral therapy; HDACi, Histone deacetylase inhibitor; HIV-1, Human immunodeficiency virus type-1; LDH, Lactate dehydrogenase; LPS, Lipopolysaccharide; LRAs, Latency-reversing agents; LTRs, Long-terminal repeats; Nef, Negative regulatory factor; NHBEs, Normal human bronchial epithelial cells; OLD, Obstructive lung diseases; pCK, Pan cytokeratin; PLWH, People living with HIV; SHIV, Simian-adapted HIV.
