# Supplementary material for: HIV-1 Productively Infects and Integrates in Bronchial Epithelial Cells
Source: Front Cell Infect Microbiol. 2021 Feb 4;10:612360. doi: 10.3389/fcimb.2020.612360 (PMC7890076; doi:10.3389/fcimb.2020.612360)
Supplement: Supplementary file 1 [file DataSheet_1.docx]

**SUPPORTING INFORMATION**

HIV-1 Productively Infects and Integrates in Bronchial Epithelial Cells

Dinesh Devadoss,^1,¶^ Shashi P. Singh,^2,¶^ Arpan Acharya,^3^ Kieu Chinh Do,^2^ Palsamy Periyasamy,^3^ Marko Manevski,^1^ Neerad Mishra,^2^ Carmen S. Tellez,^2^ Sundaram Ramakrishnan,^4^ Steven A. Belinsky,^2^ Siddappa N. Byrareddy,^3^ Shilpa Buch,^3^ Hitendra S. Chand,^1,^ ***** and Mohan Sopori ^2,^ *****

^1^ Department of Immunology and Nanomedicine, Herbert Wertheim College of Medicine, Florida International University, Miami, Florida, United States of America

^2^ Lovelace Respiratory Research Institute, Albuquerque, New Mexico, United States of America

^3^ Department of Pharmacology and Experimental Neuroscience, University of Nebraska Medical Center, Omaha, Nebraska, United States of America

^4^ Department of Surgery, University of Miami, Miami, Florida, United States of America

^¶^ These authors contributed equally to this work.

*****Corresponding Authors

Email: [msopori@lrri.org](mailto:msopori@lrri.org) (MS)

Email: [hchand@fiu.edu](mailto:hchand@fiu.edu) (HSC)

^¶^ These authors contributed equally to this work.

**Short Title**: Bronchial Epithelial Cells are HIV Targets

**
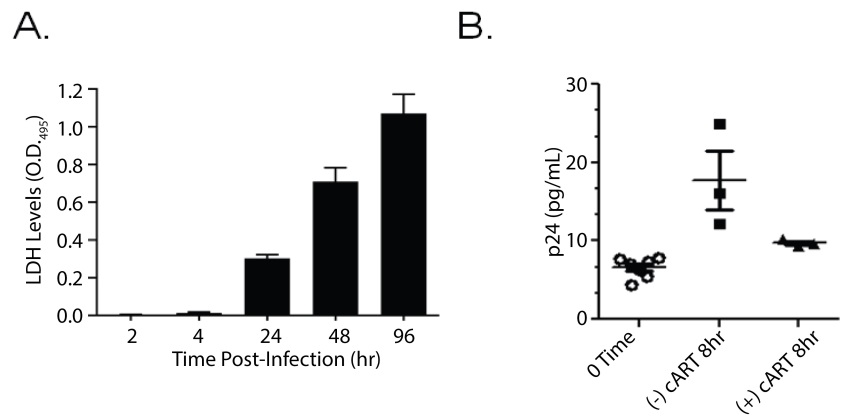
**

**Fig. S1.** **HIV-1 infection induces cytotoxicity in NHBEs, and the infection is suppressed by combined antiretroviral treatment (cART).** (**A.**) LDH release in cell culture media collected from the bottom of transwell after X4-tropic HIV-1_LAV_ infection. (**B.**) HIV-1 p24 levels after the cART (a cocktail of ritonavir, tenofovir, and emtricitabine) treatment of NHBEs. Data shown as mean ± SEM; n≥3/group. The results are representative of repeats of three different experiments with fresh batch of NHBEs.

**
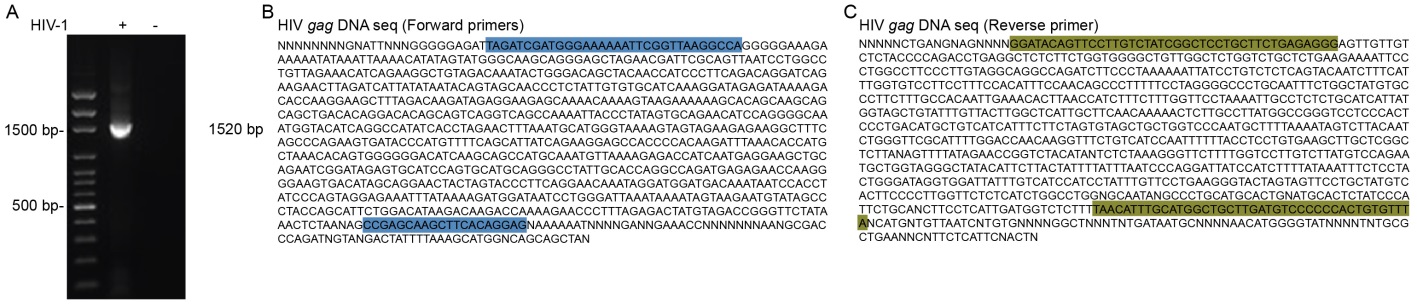
**

**Fig. S2**. **Amplification of HIV-1 gag by nested PCR and sequence analysis of the product.** HIV-1 gag nested PCR amplification was performed as described in Materials and Methods. (**A.**) Agarose gel analysis of the HIV gag PCR amplicon (1520 bp) amplified from the DNA isolated from HIV-infected NHBEs at 24 h after the infection. (**B.**) Sequence of the HIV gag DNA amplicon obtained by using the forward primers. (**C.**) Sequence of the HIV gag DNA amplicon by using the reverse primers. The forward and the reverse primer sequence of HIV-1 are highlighted in blue and green colors, respectively.

**
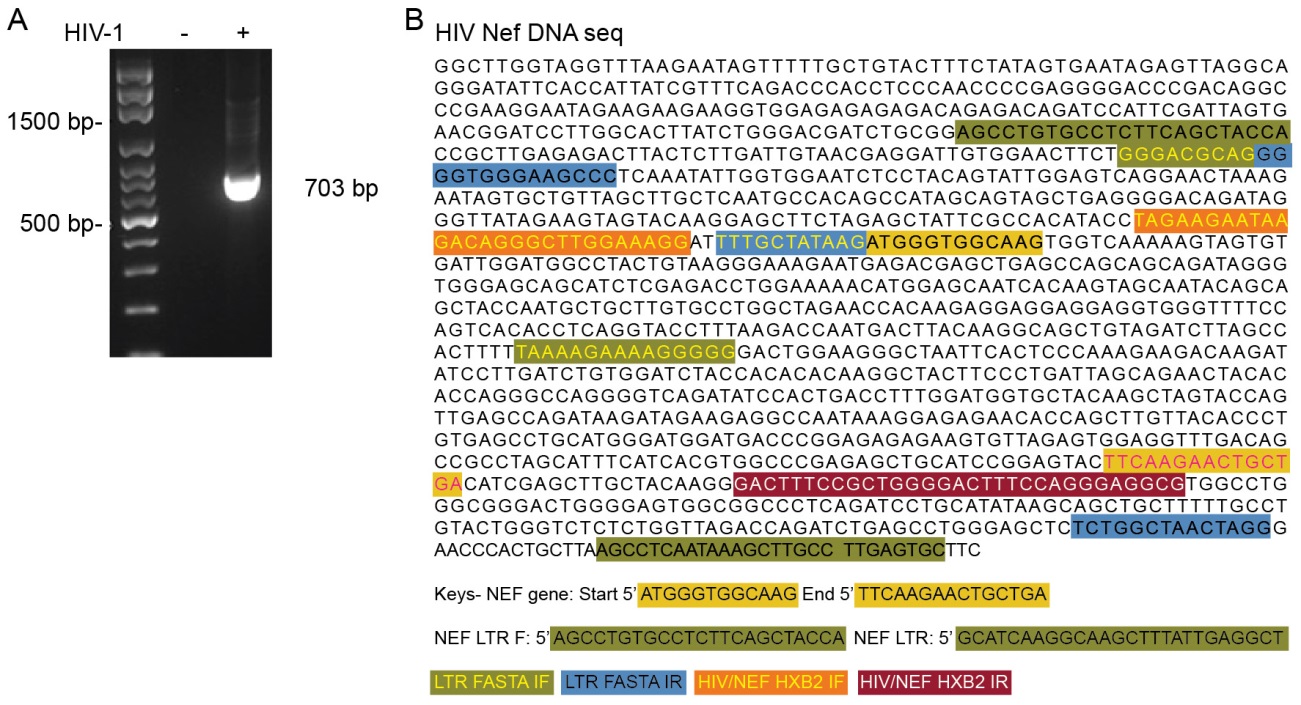
**

**Fig. S3.** **Amplification and the sequence analysis of HIV Nef DNA.** DNA was isolated from HIV-1 infected cells as described in Supplemental Figure 2. (**A.**) Agarose gel analysis of the HIV Nef PCR amplicon (703 bp) (**B.**) DNA sequence analysis of the 703 bp PCR product. The HIV Nef gene sequence 5′- and 3′- end are highlighted in yellow. The primer sequences used for Nef LTR amplification are highlighted in green, and those used for nested HIV LTR amplification are highlighted in grey with yellow font for forward primer and reverse primers are highlighted in blue. The primer sequences for the HIV Nef from HIV_HXB2_ are highlighted in black with yellow font for forward primer and are highlighted in red with white font for the reverse primer.
